# Synthesis and Antimycobacterial Activity of Symmetric Thiocarbohydrazone Derivatives against *Mycobacterium bovis* BCG

**Published:** 2013

**Authors:** Kamaleddin Haj Mohammad Ebrahim Tehrani, Farzad Kobarfard, Parisa Azerang, Maryam Mehravar, Zohreh Soleimani, Ghazaleh Ghavami, Soroush Sardari

**Affiliations:** a *Department of Medicinal Chemistry, School of Pharmacy, Shahid Beheshti University of Medical Sciences, Tehran, Iran.*; b *Phytochemistry Research Center, Shahid Beheshti University of Medical Sciences, Tehran, Iran.*; c *Drug Design and Bioinformatics Unit, Medical Biotechnology Department, Biotechnology Research Center, Tehran, Pasteur Institute, 13164, Iran.*

**Keywords:** Thiocarbohydrazone, Thiacetazone, *Mycobacterium bovis*, Antifungal, *In silico*

## Abstract

In this work, we reported the synthesis and evaluation of antimycobacterial and antifungal activity of a series of thiocarbohydrazone derivatives which are thiacetazone congeners. The target compounds were synthesized in superior yields by reacting thiocarbohydrazide with different aromatic aldehydes and methyl ketones. Compounds 8, 19 and 25 were found to be the most potent derivatives, exhibiting acceptable activity against *Mycobacterium bovis *BCG compared to thiacetazone and ethambutol as reference substances. Compounds 8, 15 and 25 exhibited the highest activity against *Candida albicans. *The most active compounds had a completely different aromatic ring system with various electronic, steric and lipophilic natures. This is understandable in light of the fact that carbohydrazone derivatives must undergo a metabolic activation step before exerting their anti-TB activity and different SAR rules govern each one of these two processes.

## Introduction

Tuberculosis (TB) is one of the most prevalent infectious diseases and based on the World Health Organization (WHO) report, 196 countries reported 2.6 million new positive TB cases in 2008 and among them, 1.78 million individuals died from TB (1). Although there are many on-going activities by pharmaceutical companies and researchers to fight the disease, there is still no great progress in this regard and emergence of resistance to the current antituberculosis drugs is widespread. 

A serious concern in antitubercular therapy is the emergence of multi-drug resistant (MDR) strains, and more recently, extensively drug-resistant (XDR) strains of *Mycobacterium tuberculosis *(2). Based on a recent WHO report, 10% of MDR cases were XDR, across all geographical regions surveyed, and thus posing the threat of an untreatable global epidemic (3, 4). Therefore, there is a need for rapid and continued progress in development of new antitubercular agents and the discovery of new cellular drug targets. 

Thiacetazone (TAC) is an economical, antitubercular, bacteriostatic drug that has been widely used in combination with other antimycobacterial agents like isoniazid (5). 

Molecular analogues of TAC, SRI-224 and SRI-286, have been synthesized and tested against *Mycobacterium avium *and found to be more effective than TAC *in-vitro *and *in-vivo *(mice) (6). In addition, our previous work on the halogenated analogues of thiacetazone led to the discovery of 4-acetamido-2-fluorobenzaldehyde thiosemicarbazone (KBF611), which was both non-toxic and highly active against *M. tuberculosis *(7). It has been shown that TAC is a prodrug that is activated by the mycobacterial monooxygenase EthA, which is also the activator of two other anti-tuberculosis drugs, ethionamide (ETH) and isoxyl (ISO) (8-10). However, the mechanism of action of TAC remains an enigma. There are indications that TAC affects mycolic acid synthesis is *M. bovis *BCG (8). 

**Figure 1:**
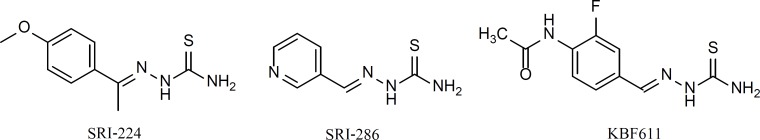
Structures of SRI-224, SRI-286 and KBF611

**Figure 2:**
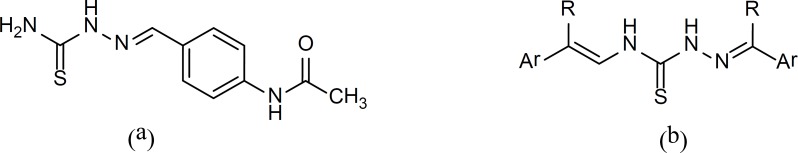
(a) Thiacetazone, (b) General structure of the new derivatives

Structure activity relationship (SAR) studies on TAC shows that the thiosemicarbazone moiety is essential for antituberculosis activity. Our previous study showed the repeated presence of such molecular fragment category in compounds library with potential antimycobacterial activity (11). Therefore, we decided to synthesize a group of compounds which could be considered as a dimerized version of TAC. In these analogs, a thiocarbohydrazone moiety is linking two aromatic systems with different substitutions, various lipophilicity, electronic and steric characteristics.

## Experimental

*General*

Melting points were obtained by an Electrothermal 9100 apparatus and are uncorrected. Infrared spectra were determined with a Perkin-Elmer 843 spectrometer. Proton nuclear magnetic resonance (1H NMR) spectra and carbon nuclear magnetic resonance (13C NMR) spectra were determined on a Bruker Avance DRX 500 MHz spectrometer and chemical shifts are reported as *δ *(ppm) in DMSO-*d*_6_ solution (0.05% v/v TMS). ESI-MS spectra were obtained using Agilent 6410 Triple Quad. LC/MS. All the compounds were analyzed for C, H, N and S on a Costech model 4010 and agreed with the proposed structures within ± 0.4% of the theoretical values. 

*General procedure for preparation of thiocarbohydrazones*

To a hot solution of thiocarbohydrazide (0.21 g, 0.002 mol) in water (6 mL) containing acetic acid (0.4 mL), selected aldehydes or methyl ketones (0.004 mol) dissolved in ethanol (15 mL) were added dropwise and the mixture was heated under reflux. After the completion of the reaction, which was determined by thin layer chromatography, the reaction was cooled to room temperature and the precipitate thus formed was filtered and rinsed with cold ethanol. In the cases that purification was needed, the crudes were recrystallized from appropriate solvents.

*Bis(benzaldehyde) thiocarbohydrazone (1)*

White powder (0.50 g, 89%): mp 195-198 °C; IR (KBr): 3303, 3175, 1606, 1542, 1525, 1254, 1130, 774, 703 cm^-1^; ^1^H NMR (DMSO-*d*_6_/500 MHz): *δ *7.42-7.48 (m, 6H, aromatic H- 3,4,5,3’,4’,5’), 7.76 (br s, 2H, aromatic H-1,5), 7.87 (br s, 2H, aromatic H-1’,5’), 8.16 (br s, 1H, azomethine H), 8.62 (br s, 1H, azomethine H), 11.58 (br s, 1H, N-H), 11.90 (br s, 1H, N-H); ^13^C NMR (DMSO-*d*_6_/125 MHz): 127.29, 128.69, 130.01, 134.12, 143.46, 148.76, 174.85; MS (ESI): 283 (M + H^+^), 305 (M + Na^+^); Anal. Calcd for C_15_H_14_N_4_S (282.36): C, 63.80; H, 5.00; N, 19.84; S, 11.36. Found: C, 63.21; H, 4.99; N, 19.76; S, 11.32.

*Bis(4-acetamidobenzaldehyde) thiocarbohydrazone (2)*

Yellow powder (0.70 g, 88%): mp 188-190 °C; IR (KBr): 3278, 3184, 1683, 1612, 1537, 1522, 1183, 849 cm^-1^; ^1^H NMR (DMSO-*d*_6_/500 MHz): *δ *2.06 (s, 6H, methyl H), 7.67 (d, *J = *6.65, 4H, aromatic H-3,5,3’,5’), 7.67 (br s, 2H, aromatic H-2,6), 7.79 (br s, 2H, aromatic H-2’,6’), 8.07 (br s, 1H, azomethine H), 8.53 (br s, 1H, azomethine H), 10.13 (s, 2H, acetamide N-H), 11.42 (br s, 1H, thiocarbamide N-H), 11.77 (br s, 1H, thiocarbamide N-H) ; ^13^C NMR (DMSO-*d*_6_/125 MHz): 24.04, 118.84, 128.00, 140.94, 142.95, 148.39, 168.53, 174.30; MS (ESI): 419 (M + Na^+^); Anal. Calcd for C_19_H_20_N_6_O_2_S (396.47): C, 57.56; H, 5.08; N, 21.20; S, 8.09. Found: C, 57.62; H, 5.11; N, 21.17; S, 8.12.

*Bis(2-fluorobenzaldehyde) thiocarbohydrazone (3)*

White powder (0.5 g, 79%): mp 196-199 °C; IR (KBr): 3288, 3122, 1612, 1537, 1518, 1252, 771 cm^-1^; ^1^H NMR (DMSO-*d*_6_/500 MHz): *δ *7.32 (m, 4H, aromatic H-4,5,4’,5’), 7.50 (m, 2H, aromatic H-3,3’), 7.98 (br s, 1H, aromatic H-6), 8.35 (br s, 1H, aromatic H-6’), 8.39 (br s, 1H, azomethine H), 8.88 (br s, 1H, azomethine H), 11.81 (br s, 1H, N-H), 12.08 (br s, 1H, N-H); ^13^C NMR (DMSO-*d*_6_/125 MHz): 115.78, 115.94, 121.65, 124.73, 126.71, 131.94, 136.08, 141.41, 159.86, 161.85, 175.17; MS (ESI): 319 (M + H^+^), 341 (M + Na^+^); Anal. Calcd for C_15_H_12_F_2_N_4_S (318.34): C, 56.59; H, 3.80; N, 17.60; S, 10.07. Found: C, 56.71; H, 3.81; N, 17.58; S, 10.02.

*Bis(3-fluorobenzaldehyde) thiocarbohydrazone (4)*

White powder (0.51, 80%): mp 206-207°C; IR (KBr): 3293, 3129, 1580, 1547, 1517, 1259, 1138, 876, 763, 689 cm^-1^; ^1^H NMR (DMSO-*d*_6_/500 MHz): *δ *7.27 (dt, *J = *8.5, *J = *2.2, 2H, aromatic H-5,5’), 7.52 (m, 3H, aromatic H-6,4,4’), 7.59 (d, *J = *7.55, 2H, aromatic H-2,2’), 7.91 (br s, 1H, aromatic H-6’), 8.15 (br s, 1H, azomethine H), 8.62 (br s, 1H, azomethine H), 11.73 (br s, 1H, N-H), 12.05 (br s, 1H, N-H); ^13^C NMR (DMSO-*d*_6_/125 MHz): 112.81, 112.99, 116.71, 116.88, 123.62, 124.64, 130.78, 136.72, 142.16, 147.57, 161.46, 163.40, 175.14; MS (ESI): 319 (M + H^+^), 341 (M + Na^+^); Anal. Calcd for C_15_H_12_F_2_N_4_S (318.34): C, 56.59; H, 3.80; N, 17.60; S, 10.07. Found: C, 56.39; H, 3.81; N, 17.53; S, 10.11.

*Bis(4-fluorobenzaldehyde) thiocarbohydrazone (5)*

White powder (0.52 g, 82%): mp 224-227 °C; IR (KBr): 3264, 3150, 1604, 1550, 1235, 1156, 843 cm^-1^; ^1^H NMR (DMSO-*d*_6_/500 MHz): *δ *7.31 (t, *J = *8.43, 4H, aromatic H-3,5,3’,5’), 7.81 (br s, 2H, aromatic H-2,6), 7.94 (br s, 2H, aromatic H-2’,6’), 8.14 (br s, 1H, azomethine H), 8.59 (br s, 1H, azomethine H), 11.61 (br s, 1H, N-H), 11.90 (br s, 1H, N-H); ^13^C NMR (DMSO-*d*_6_/125 MHz): 115.68, 115.86, 129.49, 130.73, 124.16, 147.57, 162.11, 164.08, 174.84; MS (ESI): 319 (M + H^+^), 341 (M + Na^+^); Anal. Calcd for C_15_H_12_F_2_N_4_S (318.34): C, 56.59; H, 3.80; N, 17.60; S, 10.07. Found: C, 56.82; H, 3.79; N, 17.56; S, 10.10.

*Bis(2-chlorobenzaldehyde) thiocarbohydrazone (6)*

White powder (0.67 g, 95%): mp 207°C (dec.); IR (KBr): 3301, 3192, 1603, 1539, 1478, 1247, 775 cm^-1^; ^1^H NMR (DMSO-*d*_6_/500 MHz): *δ *7.46 (m, 4H, aromatic H-4,5,4’,5’), 7.52 (m, 2H, aromatic H-3,3’), 8.06 (br s, 1H, aromatic H-6), 8.39 (br s, 1H, aromatic H-6’), 8.61 (br s, 1H, azomethine H), 8.99 (br s, 1H, azomethine H), 11.97 (br s, 1H, N-H), 12.15 (br s, 1H, N-H); ^13^C NMR (DMSO-*d*_6_/125 MHz): 127.48, 127.80, 129.90, 131.52, 133.37, 139.73, 144.63, 175.37; MS (ESI): 373 (100%), 375 (50), 377 (15%) (M + Na^+^); Anal. Calcd for C_15_H_12_Cl_2_N_4_S (351.25): C, 51.29; H, 3.44; N, 15.95; S, 9.13. Found: C, 51.11; H, 3.45; N, 15.89; S, 9.08.

*Bis(3-chlorobenzaldehyde) thiocarbohydrazone *(7)

White powder (0.67 g, 95%): mp 216-219°C; IR (KBr): 3300, 3122, 1549, 1524, 1257, 1155, 893, 798, 695 cm^-1^; ^1^H NMR (DMSO-*d*_6_/500 MHz): *δ *7.50 (s, 4H, aromatic H-4,5,4’,5’), 7.71 (s, 2H, aromatic H-6,6’), 7.79 (br s, 1H, aromatic H-1), 8.11 (br s, 2H, aromatic H-1’, azomethine H), 8.60 (br s, 1H, azomethine H), 11.78 (br s, 1H, N-H), 12.07 (br s, 1H, N-H); ^13^C NMR (DMSO-*d*_6_/125 MHz): 126.17, 126.93, 129.69, 130.61, 133.70, 136.10, 141.97, 147.20, 175.16; MS (ESI): 373 (100%), 375 (71%), 377 (17%) (M + Na^+^); Anal. Calcd for C_15_H_12_Cl_2_N_4_S (351.25): C, 51.29; H, 3.44; N, 15.95; S, 9.13. Found: C, 51.29; H, 3.42; N, 16.01; S, 9.09.

*Bis(4-chlorobenzaldehyde) thiocarbohydrazone (8)*

White powder (0.53 g, 75%): mp 205°C (dec.); IR (KBr): 3198, 3127, 1587, 1542, 1508, 1244, 825 cm^-1^; ^1^H NMR (DMSO-*d*_6_/500 MHz): *δ *7.53 (d, *J = *8.32, 4H, aromatic H-3,5,3’,5’), 7.77 (br s, 2H, aromatic H-2,6), 7.91 (br s, 2H, aromatic H-2’,6’), 8.14 (br s, 1H, azomethine H), 8.60 (br s, 1H, azomethine H), 11.67 (br s, 1H, N-H), 11.99 (br s, 1H, N-H); ^13^C NMR (DMSO-*d*_6_/125 MHz): 128.87, 129.29, 132.90, 133.25, 134.56, 142.15, 147.48, 174.95; MS (ESI): 351 (100%), 353 (50%), 355 (11%) (M + H^+^), 373 (100%), 375 (77%), 377 (20%) (M + Na^+^); Anal. Calcd for C_15_H_12_Cl_2_N_4_S (351.25): C, 51.29; H, 3.44; N, 15.95; S, 9.13. Found: C, 51.11; H, 3.44; N, 16.00; S, 9.10.

*Bis(2-bromobenzaldehyde) thiocarbohydrazone (9)*

White powder (0.73 g, 83%): mp 208 °C (dec.); IR (KBr): 3275, 3101, 1534, 1513, 1245, 1148, 685 cm^-1^; ^1^H NMR (DMSO-*d*_6_/500 MHz): *δ *7.38 (dt, *J = *7.63, *J *= 1.70, 2H, aromatic H-4,4’), 7.50 (t, *J = *7.63, 2H, aromatic H-5,5’), 7.70 (dd, *J = *8.03, *J *= 0.66, aromatic H-3,3’), 8.04 (br s, 1H, aromatic H-6), 8.37 (br s, 1H, aromatic H-6’), 8.58 (br s, 1H, azomethine H), 8.93 (br s, 1H, azomethine H), 12.03 (br s, 1H, N-H), 12.18 (br s, 1H, N-H); ^13^C NMR (DMSO-*d*_6_/125 MHz): 123.81, 127.97, 131.76, 132.69, 133.15, 142.18, 146.87, 175.41; MS (ESI): 439 (60%), 441 (100%), 443 (50%) (M + H^+^), 461 (60%), 463 (100%), 465 (50%) (M + Na^+^); Anal. Calcd for C_15_H_12_Br_2_N_4_S (440.16): C, 40.93; H, 2.75; N, 12.73; S, 7.28. Found: C, 41.01; H, 2.78; N, 12.69; S, 7.25.

*Bis(3-bromobenzaldehyde) thiocarbohydrazone (10)*

White powder (0.76 g, 86%): mp 225°C (dec.); IR (KBr): 3285, 3142, 1528, 1515, 1245, 761 cm^-1^; ^1^H NMR (DMSO-*d*_6_/500 MHz): *δ *7.42 (t, *J *= 7.79, 2H, aromatic H-5,5’), 7.63 (d, *J = *7.79, 2H, aromatic H-4,4’), 7.75 (br s, 2H, aromatic H-6,6’), 7.92 (br s, 1H, aromatic H-1), 8.12 (br s, 1H, aromatic H-1’), 8.22 (br s, 1H, azomethine H), 8,58 (br s, 1H, azomethine H), 11.79 (br s, 1H, N-H), 12.05 (br s, 1H, N-H); ^13^C NMR (DMSO-*d*_6_/125 MHz): 122.14, 126.79, 128.98, 130.77, 132.48, 136.46, 141.84, 146.90, 175.13; MS (ESI): 439 (55%), 441 (100%), 443 (56%) (M + H^+^); Anal. Calcd for C_15_H_12_Br_2_N_4_S (440.16): C, 40.93; H, 2.75; N, 12.73; S, 7.28. Found: C, 40.85; H, 2.75; N, 12.71; S, 7.26.

*Bis(4-bromobenzaldehyde) thiocarbohydrazone (11)*

White powder (0.82 g ,93%): mp 214 °C (dec.); IR (KBr): 3190, 1581, 1538, 1245, 1114, 821 cm^-1^; ^1^H NMR (DMSO-*d*_6_/500 MHz): *δ *7.67 (d, *J = *7.50, 4H, aromatic H-3,5,3’,5’), 7.67 (br s, 2H, aromatic H-2, 6), 7.83 (br s, 2H, aromatic H-2’,6’), 8.12 (br s, 1H, azomethine H), 8.58 (br s, 1H, azomethine H), 11.68 (br s, 1H, N-H), 11.99 (br s, 1H, N-H); ^13^C NMR (DMSO-*d*_6_/125 MHz): 123.38, 128.98, 129.51, 131.81, 133.22, 133.58, 142.28, 147.61, 174.94; MS (ESI): MS (ESI): 439 (40%), 441 (100%), 443 (40%) (M + H^+^), 461 (45%), 463 (100%), 465 (67%) (M + Na^+^); Anal. Calcd for C_15_H_12_Br_2_N_4_S (440.16): C, 40.93; H, 2.75; N, 12.73; S, 7.28. Found: C, 41.10; H, 2.77; N, 12.72; S, 7.26. 

*Bis(2-nitrobenzaldehyde) thiocarbohydrazone (12)*

Yellow powder (0.66 g, 89%): mp 215 °C (dec.); IR (KBr): 3263, 3105, 1506, 1339, 1237, 1112, 747 cm^-1^; ^1^H NMR (DMSO-*d*_6_/500 MHz): *δ *7.70 (dt, *J = *7.64, *J = *1.30, 2H, aromatic H-4,4’), 7.84 (t, *J = *7.64, 2H, aromatic H-5,5’), 8.08 (dd, *J = *7.64, *J = *0.90, 2H, aromatic H-3,3’), 8.18 (br s, 1H, aromatic H-6), 8.43 (br s, 1H, aromatic H-6’), 8.63 (br s, 1H, azomethine H), 8.98 (br s, 1H, azomethine H), 12.10 (br, s, N-H), 12.30 (br, s, N-H); ^13^C NMR (DMSO-*d*_6_/125 MHz): 124.69, 128.57, 130.73, 133.65, 139.26, 143.67, 148.32, 175.81; MS (ESI): 373 (M + H^+^); Anal. Calcd for C_15_H_12_N_6_O_4_S (372.36): C, 48.38; H, 3.25; N, 22.57; S, 8.61. Found: C, 48.21; H, 3.22; N, 22.65; S, 8.63.

*Bis(3-nitrobenzaldehyde) thiocarbohydrazone (13)*

Yellow powder (0.73 g, 98%): mp 222 °C (dec.); IR (KBr): 3270, 3112, 1613, 1529, 1515, 1353, 1251, 876, 743, 677 cm^-1^; ^1^H NMR (DMSO-*d*_6_/500 MHz): *δ *7.77 (t, *J *= 8, 2H, aromatic H-3,3’), 8.28 (dd, *J *= 8, *J *= 1.5, 2H, aromatic H-2,2’), 8.28 (br s, 2H, H-4,4’), 8.66 (br s, 4H, aromatic H-1,1’ and 2 azomethine H), 12.05 (br s, 1H, N-H), 12.16 (br s, 1H, N-H); ^13^C NMR (DMSO-*d*_6_/125 MHz): 121.43, 124.23, 130.30, 133.56, 135.99, 141.00, 148.29, 175.43; MS (ESI): 373 (M + H^+^); Anal. Calcd for C_15_H_12_N_6_O_4_S (372.36): C, 48.38; H, 3.25; N, 22.57; S, 8.61. Found: C, 48.34; H, 3.24; N, 22.68; S, 8.60.

*Bis(4-nitrobenzaldehyde) thiocarbohydrazone (14)*

Yellow powder (0.58 g, 78%): mp 217 °C (dec.); IR (KBr): 3284, 3153, 1545, 1523, 1377, 1327, 1260, 850 cm^-1^; ^1^H NMR (DMSO-*d*_6_/500 MHz): *δ *8.03-8.22 (m, 5H, 4 aromatic H and 1 azomethine H), 8.32 (d, *J = *8.05, 4H, aromatic H-3,5,3’,5’) 8.73 (br s, azomethine H), 12.02 (br s, N-H), 12.34 (br s, N-H); ^13^C NMR (DMSO-*d*_6_/125 MHz): 123.86, 128.17, 140.31, 146.36, 147.80, 175.50; MS (ESI): 373 (M + H^+^); Anal. Calcd for C_15_H_12_N_6_O_4_S (372.36): C, 48.38; H, 3.25; N, 22.57; S, 8.61. Found: C, 48.18; H, 3.24; N, 22.65; S, 8.63.

*Bis(pyridine-2-carbaldehyde) thiocarbohydrazone (15)*

Yellow powder (0.49 g, 86%): mp 204-207°C; IR (KBr): 3140, 1612, 1538, 1524, 1249, 1126, 912, 886, 797 cm^-1^; ^1^H NMR (DMSO-*d*^6^/500 MHz): (for the major isomer) *δ *7.42 (ddd, *J *= 7.6, *J *= 4.5, *J *= 1.0, 2H, aromatic H-4,4’), 7.90 (dt, *J *= 7.6, *J *= 1.4, 2H, aromatic H-5,5’), 7.99 (br s, 1H, aromatic H-6), 8.22 (br s, 1H, aromatic H-6’), 8.37 (br s, 1H, azomethine H), 8.61 (d, *J *= 4.5, 2H, aromatic H-3,3’), 8.64 (br s, 1H, azomethine H), 11.90 (br s, 1H, N-H), 12.22 (br s, 1H, N-H); ^13^C NMR (DMSO-*d*_6_/125 MHz): 119.65, 120.22, 124.44, 124.57, 125.05, 126.71, 136.75, 137.07, 138.56, 139.44, 143.88, 148.29, 149.55, 149.88, 151.37, 152.82, 175.49, 176.75; MS (ESI): 285 (M + H^+^), 307 (M + Na^+^); Anal. Calcd for C_13_H_12_N_6_S (284.34): C, 54.91; H, 4.25; N, 29.56; S, 11.28. Found: C, 55.00; H, 4.23; N, 29.48; S, 11.26.

*Bis(pyridine-3-carbaldehyde) thiocarbohydrazone (16)*

Pale yellow powder (0.56 g, 98%): mp 215 °C; IR (KBr): 3298, 3143, 1602, 1539, 1309, 1275, 1153, 821, 717 cm^-1^; ^1^H NMR (DMSO-*d*_6_/500 MHz): *δ *7.50 (dd, *J = *7.64, *J = *4.75, 2H, pyridine H-5,5’), 8.17 (br s, 2H, pyridine H-6,6’), 8.30 (br s, 1H, pyridine H-2), 8.62 (dd, *J = *4.75, *J = *1.46, pyridine H-4,4’), 8.66 (br s, 1H, pyridine H-2’), 8.66 (br s, 1H, azomethine H) 9.04 (br s, 1H, azomethine H), 11.78 (br s, 1H, N-H), 12.14 (br s, 1H, N-H); ^13^C NMR (DMSO-*d*_6_/125 MHz): 123.78, 129.97, 133.77, 140.63, 146.05, 148.75, 150.53; MS (ESI): 285 (M + H^+^), 307 (M + Na^+^); Anal. Calcd for C_13_H_12_N_6_S (284.34): C, 54.91; H, 4.25; N, 29.56; S, 11.28. Found: C, 54.68; H, 4.27; N, 29.53; S, 11.27.

*Bis(pyridine-4-carbaldehyde) thiocarbohydrazone (17)*

Yellow powder (0.56 g, 98%): mp 218-219°C; IR (KBr): 3214, 1597, 1562, 1511, 1261, 1131, 999, 907, 814 cm^-1^; ^1^H NMR (DMSO-*d*_6_/500 MHz): *δ *7.71 (br s, 2H, pyridine H-2,6), 7.82 (br s, 2H, pyridine H-2’,6’), 8.14 (br s, 1H, azomethine H), 8.62 (br s, 1H, azomethine H), 8.67 (d, *J = *5.85, 4H, H-3,5,3’,5’), 11.94 (br s, 1H. N-H), 12.31 (br s, 1H. N-H); ^13^C NMR (DMSO-*d*_6_/125 MHz): 121.24, 141.27, 146.84, 150.12, 175.65; MS (ESI): 307 (M + Na^+^); Anal. Calcd for C_13_H_12_N_6_S (284.34): C, 54.91; H, 4.25; N, 29.56; S, 11.28. Found: C, 54.90; H, 4.23; N, 29.51; S, 11.23.

*Bis(furan-2-carbaldehyde) thiocarbohydrazone (18)*

Yellow powder (0.46 g, 88%): mp 186 °C (dec.); IR (KBr): 3292, 3128, 1618, 1530, 1242, 1020, 944, 767, 624 cm^-1^; ^1^H NMR (DMSO-*d*_6_/500 MHz): *δ *6.6 (s, 2H, furan H-4,4’), 6.96 (br s, 2H, furan H-5,5’), 7.86 (s, 2H, furan H-3.3’), 8.05 (br s, 1H, azomethine H), 8.46 (br s, 1H, azomethine H), 11.46 (br s, 1H, N-H), 11.80 (br s, 1H, N-H); ^13^C NMR (DMSO-*d*_6_/125 MHz): 112.81, 114.17, 134.50, 139.10, 145.69, 149.71, 174.88; MS (ESI): 285 (M + Na^+^); Anal. Calcd for C_11_H_10_N_4_O_2_S (262.29): C, 50.37; H, 3.84; N, 21.36; S, 12.23 Found: C, 50.47; H, 3.82; N, 21.27; S, 12.21.

*Bis(thiophene-2-carbaldehyde) thiocarbohydrazone (19)*

Yellow powder (0.41 g, 70%): mp 196 °C (dec.); IR (KBr): 3294, 3114, 1589, 1543, 1526, 1258, 1224, 1130, 707 cm^-1^; ^1^H NMR (DMSO-*d*_6_/500 MHz): *δ *7.15 (dd, *J = *4.86, *J = *3.63, 2H, thiophene H-4,4’), 7.48 (br s, 2H, thiophene H-5,5’), 7.70 (d, *J = *4.86, 2H, thiophene H-3,3’), 8.37 (br s, 1H, azomethine H), 8.68 (br s, 1H, azomethine H), 11.40 (br s, 1H, N-H), 11.76 (br s, 1H, N-H); ^13^C NMR (DMSO-*d*_6_/125 MHz): 127.95, 129.22, 130.99, 138.51, 143.98, 173.97; MS (ESI): 295 (M + H^+^), 317 (M + Na^+^); Anal. Calcd for C_11_H_10_N_4_S_3_ (294.42): C, 45.87; H, 3.42; N, 19.03; S, 32.67. Found: C, 45.03; H, 3.40; N, 19.09; S, 32.76.

*Bis(acetophenone) thiocarbohydrazone (20)*

Recrystallized from ethanol; yellow powder (0.39 g, 63%): mp 188 °C (dec.); IR (KBr): 3282, 3191, 1511, 1478, 1125, 791, 683 cm^-1^; ^1^H NMR (DMSO-*d*_6_/500 MHz): *δ *2.38 (s, 6H, CH_3_), 7.45 (m, 6H, aromatic H-3,4,5,3’,4’,5’), 7.88 (d, *J *= 7.80, 4H, aromatic H-2,6,2’6’), 10.96 (br s, 2H, NH); MS (ESI): 311 (M + H^+^); Anal. Calcd for C_17_H_18_N_4_S (310.42): C, 65.78; H, 5.84; N, 18.05; S, 10.33. Found: C, 65.60; H, 5.83; N, 18.01; S, 10.36.76.

*Bis (N-(4-acetylphenyl) acetamide) thiocarbohydrazone (21)*

Recrystallized from ethyl acetate; yellow powder (0.23 g, 54%): mp 220 °C (dec.); IR (KBr): 3254, 3380, 3245, 1667, 1511, 1499, 1229, 850 cm^-1^; ^1^H NMR (DMSO-*d*_6_/500 MHz): *δ *2.06 (s, 6H, ‒C(O)C*H*_3_), 2.34 (s, 6H, ‒C(N)C*H*_3_), 7.64(d, *J *= 8.69, 4H, aromatic H-3,5,3’,5’), 7.82 (d, *J *= 8.69, 4H, aromatic H-2,6,2’,6’), 10.10 (s, 2H, NH), 10.76 (br s, 2H, NH); MS (ESI): 425 (M + H^+^); Anal. Calcd for C_21_H_24_N_6_O_2_S (424.52): C, 59.41; H, 5.70; N, 19.80; O, 7.54; S, 7.55. Found: C, 59.31; H, 5.71; N, 19.86; S, 32.73.

*Bis (4’-fluoroacetophenone) thiocarbohydrazone (22)*

Recrystallized from ethanol; yellow powder (0.37 g, 53%): mp 212-215 °C; IR (KBr): 2365, 3150, 1595, 1487, 1225, 847, 834 cm^-1^; ^1^H NMR (DMSO-*d*_6_/500 MHz): *δ *2.36 (s, 6H, CH_3_), 7.28 (t, *J *= 8.80, 4H, aromatic H-3,5,3’,5’), 7.94 (dd, *J *= 8.80, *J *= 5.6, 4H, aromatic H-2,6,2’,6’), 10.80 (br s, 2H, NH); MS (ESI): 347 (M + H^+^); Anal. Calcd for C_17_H_16_F_2_N_4_S (346.40): C, 58.94; H, 4.66; N, 16.17; S, 9.26. Found: C, 59.01; H, 4.65; N, 16.22; S, 9.25.

*Bis(4’-cloroacetophenone) thiocarbohydrazone (23)*

Recrystallized from ethanol; yellow powder (0.45 g, 59%): mp 213-216 °C; IR (KBr): 3290, 1606, 1484, 1319, 1230, 1138, 830, 767 cm^-1^; ^1^H NMR (DMSO-*d*_6_/500 MHz): *δ *2.36 (s, 6H, CH_3_), 7.51 (d, *J *= 8.61, 4H, aromatic H-3,5,3’,5’), 7.91 (d, *J *= 8.61, 4H, aromatic H-2,6,2’,6’), 10.87 (s, 2H, NH); MS (ESI): 379, 381, 383 (M + H^+^); Anal. Calcd for C_17_H_16_Cl_2_N_4_S (379.31): C, 53.83; H, 4.25; N, 14.77; S, 8.45. Found: C, 53.95; H, 4.25; N, 14.73; S, 8.47.

*Bis(4’-bromoacetophenone) thiocarbohydrazone (24)*

Yellow powder (0.85 g, 92%): mp 209°C (dec.); IR (KBr): 3290, 3183, 1596, 1509, 1477, 1229, 1008, 853 cm^-1^; ^1^H NMR (DMSO-*d*_6_/500 MHz): *δ *2.35 (s, 6H, CH_3_), 7.64 (d, *J *= 8.50, 4H, aromatic H-3,5,3’,5’), 7.83 (d, *J *= 8.50, 4H, aromatic H-2,6,2’,6’), 10.88 (s, 2H, NH); MS (ESI): 467, 469, 471 (M + H^+^); Anal. Calcd for C_17_H_16_Br_2_N_4_S (468.21): C, 43.61; H, 3.44; N, 11.97; S, 6.85. Found: C, 43.70; H, 3.43; N, 11.93; S, 6.84.

*Bis (2-acetylpyridine) thiocarbohydrazone (25)*

Recrystallized twice from ethanol; yellow powder (0.32 g, 51%): mp 163-165°C; IR (KBr): 3192, 1583, 1551, 1463, 1227, 1118, 855 cm^-1^; ^1^H NMR (DMSO-*d*_6_/500 MHz): *δ *2.47 and 2.49 (each s, 3H, CH_3_), 7.44 (dd, *J *= 7.4, *J *= 4.8, 2H, pyridine H-5,5’), 7.90 (t, *J *= 7.8, 2H, pyridine H-4,4’), 8.19 (d, *J*=7.8, 2H, pyridine H-3,3’), 8.63 (d, *J *= 4.8, 2H, pyridine H-6,6’), 11.00 (br s, 2H, NH).; MS (ESI): 313 (M + H^+^); Anal. Calcd for C_15_H_16_N_6_S (312.39): C, 57.67; H, 5.16; N, 26.90; S, 10.26 Found: C, 57.54; H, 5.16; N, 26.96; S, 10.23.

*Bis(4-acetylpyridine) thiocarbohydrazone (26)*

Yellow powder (0.40 g, 64%): mp 233-236 °C; IR (KBr): 3312, 3147, 1593, 1510, 1486, 1217, 1111, 815cm^-1^; ^1^H NMR (DMSO-*d*_6_/500 MHz): *δ *2.39 (s, 6H, CH_3_), 7.82 (d, *J*=4.60, 4H, aromatic H-2,6,2’,6’), 8.65 (d, *J*=4.60, 4H, aromatic H-3,5,3’,5’), 11.10 (s, 2H, NH); MS (ESI): 313 (M + H^+^); Anal. Calcd for C_15_H_16_N_6_S (312.39): C, 57.67; H, 5.16; N, 26.90; S, 10.26. Found: C, 57.50; H, 5.18; N, 26.82; S, 10.25.

*In-vitro evaluation of anti-mycobacterial activity*

To evaluate the antimycobacterial activity of the compounds, the broth microdilution method (12) against *M. bovis *BCG (1173P2) was used. The test compounds were initially dissolved in DMSO to give a concentration of 1 or 2 mg/L. All wells of microplates received 100 μL of freshly prepared Middle broke 7H9 medium (Himedia, India), except the first column. Two hundred μL of distilled water was added to the first column of 96 well plates to minimize the evaporation of the medium in the test wells during the incubation. Then, 100 μL of test compounds with desired concentrations (1000 or 2000 μL) were added to the wells of the first row (each concentration was assayed in duplicate) and serial dilution was made from the first row to the last. Microbial suspension of BCG (1173P2) (100 μL), which had been prepared with standard concentration of 0.5 Mcfarland and diluted with 1:10 proportion by the distilled water, was added to all test wells. Plates were then sealed and incubated for 4 days at 37°C. After that, 12 μL Tween 80 10% and 20 μL Alamar blue 0.01% (Himedia, India) were added to each test well. The results were assessed after 24 and 48 h. A blue color was interpreted as no bacterial growth, and color change to pink was scored as bacterial growth. Wells with a well-defined pink color were scored as positive for growth. The MIC (Minimal Inhibition Concentration) was defined as the lowest drug concentration, which prevented a color change from blue to pink. Ethambutol (Irandaru, Tehran) and thiacetazone were used as positive control and DMSO as negative one.

*In-vitro evaluation of antifungal activity*

The antifungal activity was evaluated by the modified antimicrobial susceptibility testing based on NCCLS M27-A method (13) against *Candida albicans *ATCC 10231. The compounds were dissolved in dimethyl sulfoxide (DMSO) to reach the concentration of 0.5 or 1 mg/mL. The absorbance was read at 530 nm for fungi inoculums to reach the suitable density of microorganisms in order to yield the desired transmittance that is 75-77% equal to a particular fungal density. Working fungal culture was prepared from the stock fungal culture, 1:1000 dilution with broth (*e.g. *10 μL stock fungal culture: 10 mL broth). Sabouraud maltose broth (DIFCO, Becton, Dickinson, USA) was used as the growth medium. Broth (100 μL) was added to each well of a 96-well microplate and then 40 μL of compounds and 60 μL broth were added to well (A), then a solution (100 μL) serially diluted from well (A) by taking 100 μL into (B) was obtained. This two-fold dilution was continued down the plate and 100 μL from the last well (H) was discarded. Then, all the wells were filled with 100 μL of working fungal culture. Itraconazole and Amphotericin B were used as a reference in the antifungal test. For this experiment and the following controls were prepared wells containing serial dilution of DMSO and itraconazole only. The plate was covered and incubated at 37°C for 24-48 h. The MIC values were obtained by reading the concentration of the well with no growth.

*Brine shrimp toxicity study *

Brine shrimp lethality bioassay (14-17) was performed to investigate the toxicity of compounds 8, 12, 19 and 25 which showed the highest antimycobacterial activity. Dried cysts (1 g cyst per liter) of brine shrimp (*Artemia salina*) were hatched in a bottle containing artificial sea water (3.5% (w/v) marine salts/distilled water) at 28-30°C with strong aeration (flow rate of 7 L/ min), under a continuous light regime (1600 lux) for 30-35 h. Subsequently, the newly hatched brine shrimp larvae (nauplii) were separated from the remaining cysts and collected with a pipette from the lighted side and concentrated in Petri dishes to be immediately used for bioassay. Assays were performed in 24-well flat test plates (Orange Scientifique, Belgium). Acetone 100% (Merck, Germany) was used for the preparation of different concentrations (1000, 100, 10 and 1 μg/mL) of tested compounds, in triplicates. Each well of treated groups became exposed to several concentration of acetone solution of compounds in the basic salt medium (3.5% (w/v) marine salts/ distilled water in addition to polyethylene glycol (PEG) 6000 (Merck, Germany) 1.2%, while control groups only received basic salt medium. Gallic acid (Merck, Germany) was utilized as positive control. After the evaporation of vehicle solvent, 10 fresh nauplii were introduced to each well and the plates were placed on a shaker with 40 rpm to be aerated at room temperature. After 24 h, the numbers of survivors (larvae were considered dead if they did not exhibit any internal or external movement during several seconds of observation) were counted by microscope AC 230V, 50 Hz (Sairan, Iran) and recorded to determine the corrected mortality via the following formula: 

Corrected mortality (%) = [(Mm_ct_)_t_ - (Mm_ct_)_c_ / 100 - (Mm_ct_)_c_] × 100 

Here: Mm_ct_ (mortality of individuals at time t%) = [N_Mm_ (number of died individuals) / N_0_ (initial number of living individuals in every test well at the beginning of the test)] × 100 

(Mm_ct_)_t_ = calculated Mm_ct_ for treated test wells 

(Mm_ct_)_c_ = calculated Mm_ct_ for control test wells 

Based on the calculated corrected mortality, relevant 50% lethality doses (LD_50_)s with 95% confidence intervals were estimated by GraphPad Prism 5.0 (2007) for each tested compound. 

*In silico calculation of the physicochemical properties*

The Clog*P *values were obtained through server tool available at (www.organic-chemistry. org). The other properties were measured by Toolkit for Estimating Physicochemical Properties of Organic Compounds (V. 1.0, 1999). The molecules in sdf format were made into database using EdiSDFd (V. 5.03, 2010). After that the energy minimization was performed through MMFF94 Energy Local Minimum, the group matching for the ranking based on key points in thiacetazone pharmacophores was measured in LigandScout (V.3.0, 2011). Dipole moment was calculated in Chem3D module of Chembiooffice 12.0 package (2010). Energy minimization was first run by MM2, and then dipole was calculated by GAMESS interface. 

## Results and discussion

*Chemistry*

**Figure 3:**
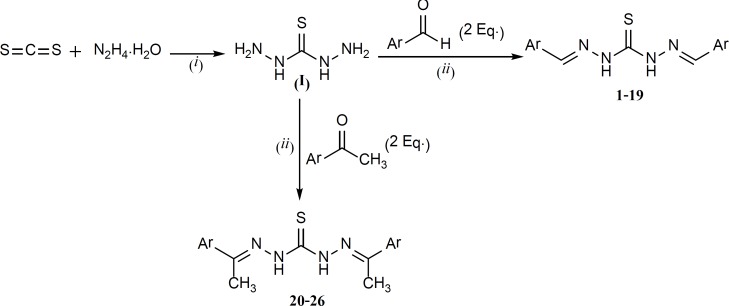
The route for the preparation of thiocarbohydrazones; reaction conditions: (*i*) H_2_O, reflux. (*ii*) H_2_O/Ethanol and acetic acid (catalytic amount), reflux.

The overall synthetic route is summarized in Figure 3. 

Thiocarbohydrazide was the key intermediate which was readily synthesized based on the method reported in the literature (18) (30% yield). The next step was a Schiff base formation which was performed by the reaction between one mole equivalent of thiocarbohydrazide and two mole equivalents of different aromatic aldehydes or methyl ketones. This reaction was quite convenient with satisfying yields ranging from 55 to 90%. 

The IR, ^1^H-NMR and ^13^C-NMR spectra were consistent with the desired derivatives which were also confirmed by electrospray (ESI) mass spectrometry. 

**Figure 4:**
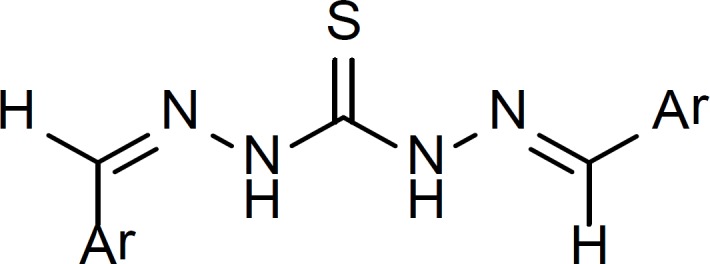
General structure for compounds 1, 3, 4, 5, 7, 8, 10, 11, 16, 18

Several of the synthesized compounds (1, 3, 4, 5, 7, 8, 10, 11, 16, 18) showed two olefinic carbons in ^13^C-NMR spectra suggesting the existence of two diastereomeric carbazone moieties in these compounds (Figure 4). 

**Figure 5:**
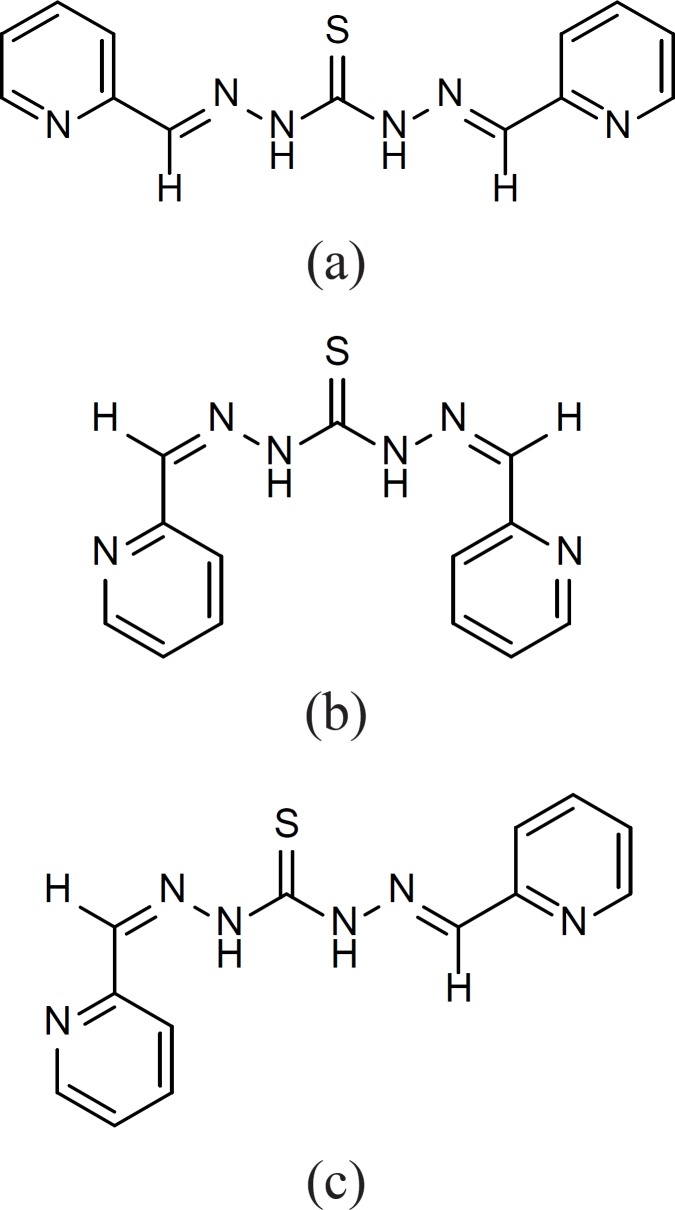
Compound 15 as different isomers

Compounds 2, 6, 9, 12, 13, 14, 17 and 19 on the other hand showed symmetric structures and no diastereomeric moieties were recognized in their ^13^C-NMR spectra. Only compound 15 showed the existence of the mixture of different isomers a-c (Figure 5). 

^1^H-NMR spectrum of this compound shows two series of peaks for the pyridine ring confirming the existence of both a and b isomers. Comparing the integration of these two series of peaks suggests the existence of 2:1 ratio for the isomers. Besides, these two series of peaks, the presence of one extra triplet and one extra doublet peak in ^1^H-NMR suggest the existence of c isomer in the mixture. Attempts to separate different isomers of compound 15 were not successful probably due to the interconversion of the isomers during the purification process. 

**Figure 6:**
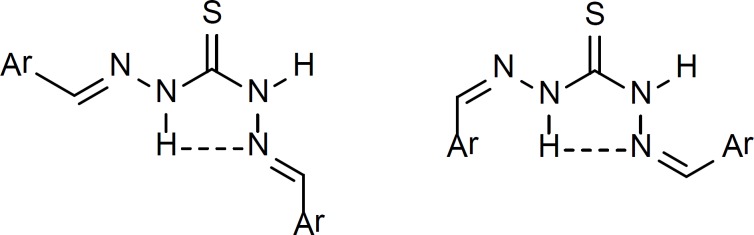
Existence of possible intramolecular H-bond in both E and Z isomers. H-bonds are shown by dashed lines.

Another plausible explanation for the above mentioned extra olefinic hydrogen and carbon signals in ^1^H NMR and ^13^C NMR spectra could be the possibility of the formation of intramolecular hydrogen bond between the imine nitrogen and thioamide N-H (Figure 6). 

This intramolecular H-bond is possible in both Z and E isomers of thiocarbazone compounds. Involvement of the imine nitrogen in the intramolecular H-bond will cause a difference in electronic environment of the two olefinic hydrogens and carbons in thiocarbazone compounds and therefore they appear at different chemical shifts. The existence of intramolecular H-bond of this type has been proven by X-ray crystallography in crystals of thiosemicarbazone derivatives before (19, 20). 

^1^H NMR spectra obtained for the synthesized derivatives in the present study suggest the existence of the same type of H-bonding in dimethylsulfoxide as the solvent. 

**Figure 7:**
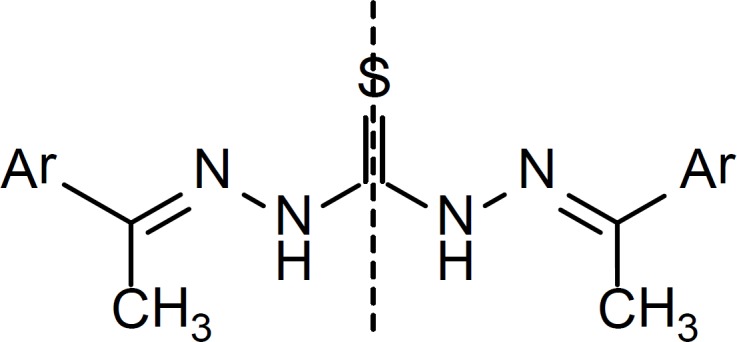
Proposed symmetric structure for compounds 20-26 based on ^1^H NMR spectra

However, in the case of the thiocarbohydrazones of methyl ketones (20-26), ^1^H NMR data suggests a symmetric structure in which the C=S bond is located on the symmetry line of the molecule (Figure 7). 

In the ^1^H NMR spectra of these derivatives, each proton in one side of the symmetry line is the chemical shift equivalent of the corresponding proton in the other side. This could be for the reason that the bulky methyl groups limit the flexibility of the molecule needed to adopt the conformation suitable for intramolecular hydrogen bonding as shown in Figure 6 and the molecule seems to prefer a symmetric conformation. 

*Biological activity*

The *in-vitro *antimycobacterial and antifungal assays were performed at Pasteur Institute (Tehran, Iran). The antimycobacterial activity of the compounds was measured by the broth microdilution method (12) against *M. bovis *BCG (1173P2) and ethambutol and thiacetazone were used as standard controls. The *in-vitro *antifungal activity evaluation was performed by the modified antimicrobial susceptibility testing based on NCCLS M27-A method (13). *Candida albicans *ATCC 10231 was used as test strain and itraconazole and amphothericin B as standard controls. The MIC (Minimal Inhibition Concentration) was defined as the lowest concentration which could inhibit the mycobacterial or fungal growth. The detailed procedure is described in the experimental section. 

The synthesized derivatives and their antimycobacterial activities are listed in Table 1.

**Table 1:** Antimycobacterial activity data of the synthesized compounds

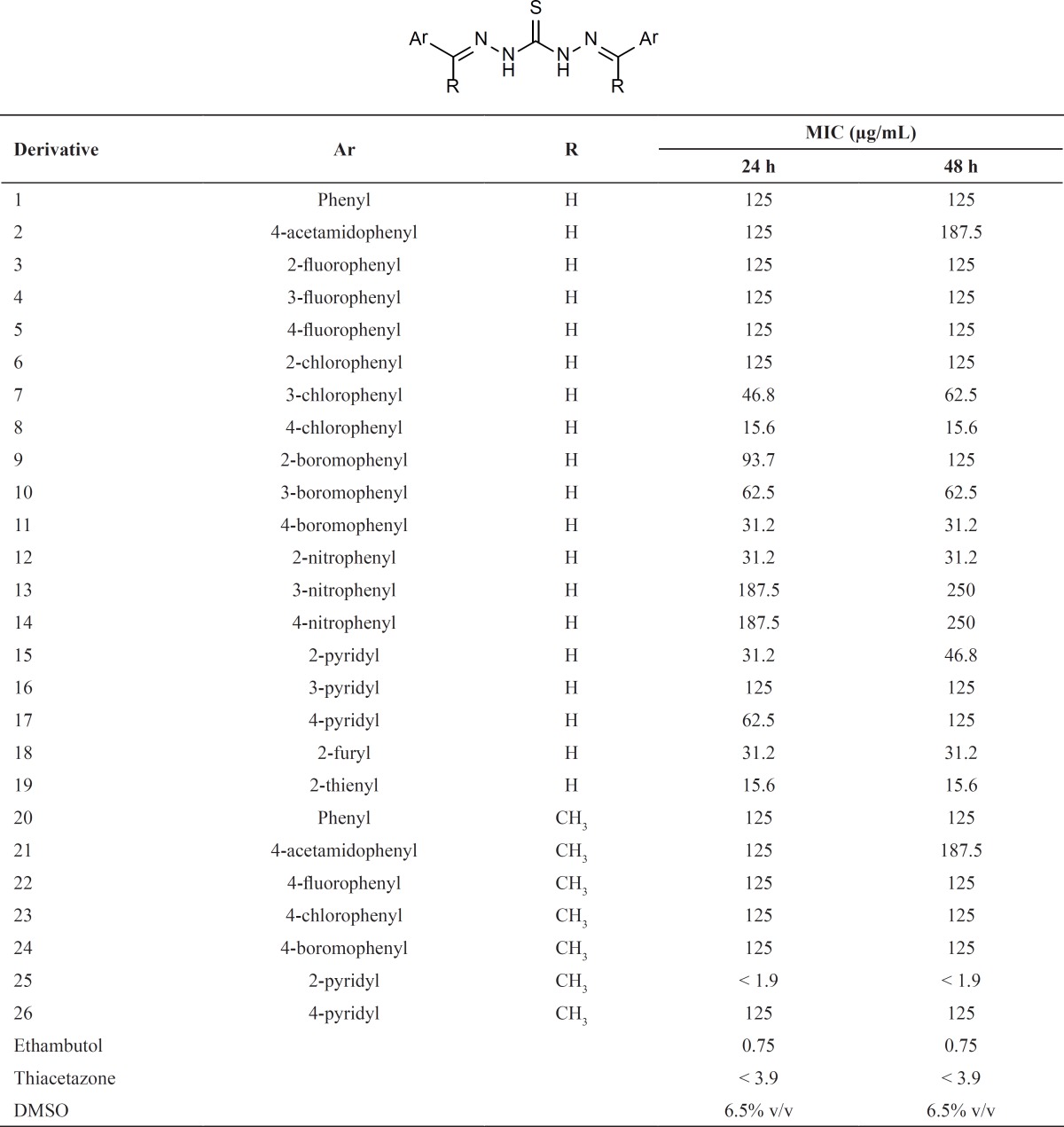

 The most active compounds are 25 (MIC < 1.9 μg/mL), 19 and 8 with MICs of 15.6 μg/mL. The MICs for standard controls (thiacetazone and ethambutol) were obtained less than 3.9 and 0.75 μg/mL, respectively. Compounds 11, 12, 15 and 18 had acceptable activities (MIC = 31.3 μg/mL) among the other analogs. Calculation of general physicochemical properties for compounds 1-26 *i.e. *Clog*P*, dipole moment, and log*P *(O/W) are shown in Table 2. 

**Table 2:** The predicted properties and the pharmacophore fitness score data of synthesized compounds.

**Compound**	**Dipole (Debye)**	**log** ***P *** **(O/W)** ^1^	**Clog** ***P*** ^2^	**Pharmacophore score** ^3,5^
1	6.77	3.00	3.72	68.57
2	6.06	1.84	2.93	88.62
3	5.51	3.28	3.84	37.54
4	4.03	3.28	3.84	58.15
5	6.07	3.28	3.84	68.67
6	5.41	4.24	4.95	57.97
7	4.33	4.24	4.95	68.62
8	6.12	4.24	4.95	68.68
9	4.65	4.78	5.12	68.44
10	4.39	4.78	5.12	57.98
11	6.23	4.78	5.12	68.68
12	4.54	2.92	3.46	57.53
13	2.43	2.92	3.46	68.22
14	7.64	2.92	3.46	67.92
15	5.52	1.25	1.57	37.09
16	4.28	0.78	1.57	56.79
17	5.91	0.78	1.57	68.75
18	7.42	3.28^4^	1.93	57.98
19	7.55	2.74^4^	3.41	68.75
20	7.13	2.92	4.19	68.66
21	7.82	1.75	3.40	88.17
22	7.44	3.19	4.31	68.73
23	7.07	4.16	5.41	68.72
24	7.02	4.70	5.58	68.74
25	8.51	1.17	2.24	68.48
26	6.97	0.70	2.03	68.61
Thiacetazone	8.53	0.45	0.90	89.00

^1^Obtained by Toolkit application**; **^2^obtained from organic-chemistry.org server; ^3^measured based on thiacetazone; ^4^measured by GCM of Viswanadhan method; ^5^Since the data in this column is calculated based on thiacetazone structural features, the data is only used to study the structure-antitubercular activity relationship related to antimycobacterial property of the compounds

Attempts to correlate these properties to the activity of the compounds reveal some relationship. Barry *et al. *(21) have identified that the optimum log*P *range for antimycobacterial property is 1.3-4.1. The calculated log*P *values indicated in Table 2 shows that compounds 15, 18, 19 and 25 with strong activity fall into this category. However, as indentified in that study, lipophilic character alone in regard to the molecular bioactivity may not correlate well with increased antimicrobial activity. On the other hand, *in-vitro *studies of thiosemicarbazone analogs perhaps offer the clearest demonstration of activity versus optimal hydrophobicity (22). For example, this study afforded the correlation of log*P *and the type of *Mycobacterium *tested in the bioassay. They indicated that for *M. tuberculosis *and a variety of slow-growing mycobacteria, the optimum log*P *value was 4, whereas for a fast-growing strain such as *M. smegmatis*, the optimum was 3. Log*P *in higher range contributes to a shift from optimum needed values that may possibly prevent the compound to reach the biological target points due to absorption to the cellular lipids. 

**Figure 8:**
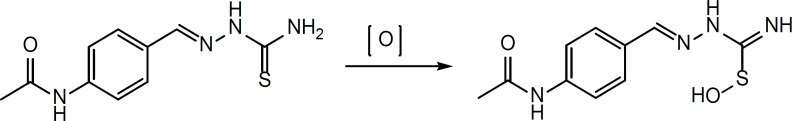
Metabolic activation of thiacetazone by a flavin-containing monooxygenase in *Mycobacterium.*

Many physicochemical factors can in a complicated manner affect the activity of antimycobacterial agents (23). Dipole moment is among the factors that influence the activity of the compounds. In general, the lower the dipole moment, the better would be the antimycobacterial activity. This is particularly indicated in compounds 12 and 15. However, a general weak activity is seen in fluorinated derivatives, while in the chlorinated and brominated compounds, the bioactivity is mainly seen in 4-substituted ones in accordance with the pharmacophore score increase. Among these, low dipole value is seen among the less active compounds that matches the pattern of increase in pharmacophore score. Our results for the possible effect of the minimized shape of molecules compared to thiacetazone provide certain affirmative clues on such correlations. In this regard, compounds 8, 11, 19 and 25 show high spatial pharmacophore arrangement after the alignment with thiacetazone (Table 2). Although there are very high pharmacophore score values for compounds 2 and 21, as the highest, these compounds do not seem to present other suitable values, such as lipophilicity (log *P *(O/W)) and in particular dipole moment, in the optimum needed range.

Thiacetazone is a second-line drug for the treatment of tuberculosis. From the chemical standpoint, thiacetazone belongs to thiocarbonyl derivatives and it has long been known that these derivatives could be prodrugs that have to be converted to their active forms by mycobacterial enzymes (24). In 2000, two independent research groups reported that EthA is involved in the activation of thiocarbonyl drugs in both *M. tuberculosis *and *M. leprae *and resistance of *M. tuberculosis *to thiacetazone involves mutations in EthA (20, 22). These findings suggest that compounds 1-26 could also be considered as prodrugs which need to be converted to their active S-oxide form by a flavin containing monooxygenase in mycobacteria. The active forms will then interfere with some essential and vital processes in *Mycobacterium *cell such as cyclopropanation of mycolic acid (25).

Considering the fact that antimycobacterial effect of thiocarbohydrazone derivatives 1-26 depends on two independent processes, *i.e. *metabolic activation and inhibition of cyclopropanation of mycolic acid in *Mycobacterium*, it is not surprising that no decisive structure-activity relationship could be concluded for these compounds. 

Besides the antitubercular activity, the antifungal activity of the new derivatives against *C. albicans *is noticeable. Compound 25 exhibited the highest activity (MIC < 3.25 μg/mL) against *C. albicans*. The derivatives with acceptable activity include compounds 8 and 15 (MIC = 15.63 μg/mL) and 19 (MIC = 62.5 μg/mL) (Table 3). 

**Table 3:** Antifungal activity of the synthesized compounds against *C. albicans *using broth dilution method.

**Derivative **	**MIC (μg/mL) **	
	**(24 h) **	**(48 h) **
1	500	1000
2	1000	> 1000
3	500	1000
4	500	1000
5	1000	> 1000
6	125	500
7	500	1000
8	15.625	31.25
9	1000	1000
10	250	1000
11	62.25	125
12	1000	1000
13	500	1000
14	500	1000
15	15.625	31.25
16	500	1000
17	1000	1000
18	250	500
19	62.5	125
20	500	1000
21	500	1000
22	500	1000
23	500	1000
24	1000	1000
25	< 3.25	< 3.25
26	500	1000
Itraconazol	0.5	2
Amphotericin B	< 0.25	0.25
DMSO	10% v/v	10% v/v

Other derivatives showed weak (if any) activity. The important point is that compounds which are active against *C. albicans *have excellent or acceptable antitubercular activity as well. In this regard, compound 25 is superior to all, since it is the most active compound against both tested organisms. This finding is valuable in order to conduct the mechanistic studies in the future on the targets that play vital rules in both organisms and may lead to revealing less noticed metabolic pathways, in which the newly synthesized derivatives may interfere. 

Determining the cytotoxicity properties of given drug candidates is crucial to their fate in lead identification and the following phases of drug discovery process. In recent years, there has been a number of toxicity tests developed in which the response has been demonstrated in invertebrates. Among these tests, the brine shrimp lethality bioassay (14, 15, 16 and 26) enjoys the advantages of being inexpensive, reproducible, conveniently handled and environmentally relevant which make it a practical method for preliminary assessment of toxicity. By carefully controlling the factors such as temperature, composition, salinity of the medium and the age of the larvae, the obtained result will be accompanied by satisfactory repeatability. Though insufficient to deduce the mechanism of action, the brine shrimp bioassay is vastly used as primary toxicity assessment of natural and synthetic leads (15). It has been demonstrated that the nature of the systems in brine shrimp which responds to drugs appears to be similar to the mammalian systems. As s result, this bioassay has been suggested for screening biological activities.

**Figure 9:**
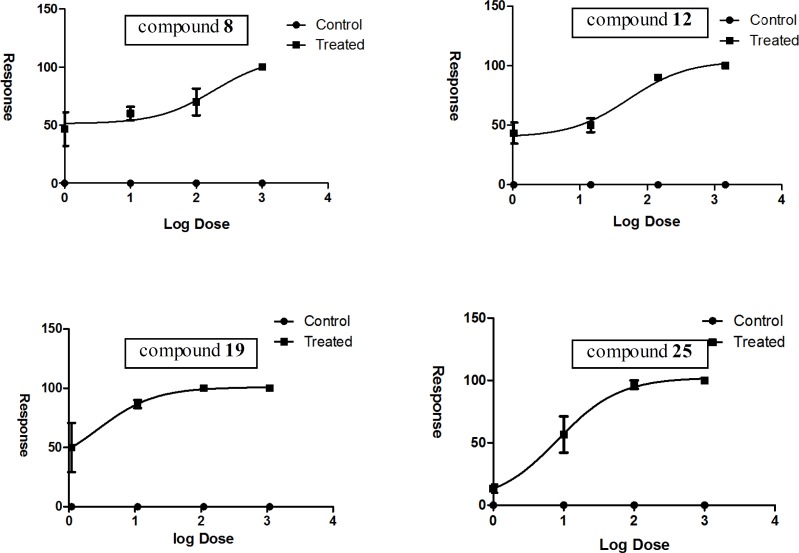
Non-linear regression curve for brine shrimp toxicity of compounds 8, 12, 19 and 25

In this study, the acute toxicity of four of the synthesized derivatives with highest antimycobacterial potency in addition to considering their diversity, were investigated by means of the *A. salina *short-term bioassay at Pasteur Institute (Tehran, Iran). The related LD_50_s (Figures 9) were calculated based on analytical method non-linear regression (dose response inhabitation). The analyzed data demonstrated that the selected derivatives caused lethality to nauplii with LD_50_ values ranging from 2.69 to 188.10 μg/mL (Table 4).

**Table 4:** Data obtained by *Artemia *lethality bioassay

**Derivative**	**LD**_50_ ** (μg/mL)**	**LD**_50_ ** (95% Confidence Intervals)**	**SI**
8	188.10	12.90 to 2741.00	12.04
12	53.22	16.07 to 176.20	1.71
19	2.69	0.06 to 122.80	0.17
25	8.23	2.94 to 23.01	4.32
Gallic acid	23.84	9.78 to 58.13	

To explore the selectivity of antimycobacterial potency for each tested compound, a selectivity index (SI) was determined by dividing LD_50_ to MIC. The range of SIs thus calculated was between 0.17 to 12.04. Based on given findings, it may indeed be true that tested compounds 8, 12 and 25 with SIs 12.04, 1.71 and 4.32 have discriminating toxicity against *Mycobacterium bovis BCG *in comparison to eukaryotic organism. There would be no doubt that considering SIs for candidate compounds can lead to presenting a set of new antimycobacterial candidates with minor undesirable side effects for further investigations. In this study, data suggest that compound 25 is a potent anti-mycobacterial and antifungal compound with excellent MICs and acceptable selectivity index which shows its safety on the tested eukaryotic organism. Further focused analog synthesis and antitubercular/antifungal activity studies are underway for compounds 8, 19 and 25 which may lead to new derivatives with enhanced efficiency against *M. tuberculosis *and fungal species*.*

## Conclusion

Amongst the 26 synthesized compounds, some of them exhibited a pronounced activity against *M. bovis *BCG and *C. albicans *with MIC values comparable with that of the standard drugs. These derivatives can be considered as starting points for further modifications to reach compounds with promising anti-tubercular and antifungal activity to enter into other complicated assays involving mechanism of action, *in-vivo *tests and perhaps clinical trials.
